# Does Cysteine Rule (CysR) Complete the CendR Principle? Increase in Affinity of Peptide Ligands for NRP-1 Through the Presence of N-Terminal Cysteine

**DOI:** 10.3390/biom10030448

**Published:** 2020-03-13

**Authors:** Anna K. Puszko, Piotr Sosnowski, Françoise Raynaud, Olivier Hermine, Gérard Hopfgartner, Yves Lepelletier, Aleksandra Misicka

**Affiliations:** 1 Faculty of Chemistry, University of Warsaw, Pasteura 1, 02-093 Warsaw, Poland; apuszko@chem.uw.edu.pl; 2 Department of Inorganic and Analytical Chemistry, University of Geneva, 24 Quai Ernest Ansermet, CH-1211 4 Geneva, Switzerland; piotr.sosnowski@unige.ch (P.S.); gerard.hopfgartner@unige.ch (G.H.); 3 Imagine Institute, Université de Paris, 24 boulevard Montparnasse, 75015 Paris, France; francoisechamp94@gmail.com (F.R.); ohermine@gmail.com (O.H.); 4 INSERM UMR 1163, Laboratory of Cellular and Molecular Basis of Normal Hematopoiesis and Hematological Disorders: Therapeutical Implications, 24 boulevard Montparnasse, 75015 Paris, France; 5 CNRS ERL 8254, 24 boulevard Montparnasse, 75015 Paris, France; 6 Department of Neuropeptides, Mossakowski Medical Research Centre, Polish Academy of Sciences, Pawinskiego 5, 02-106 Warsaw, Poland

**Keywords:** Neuropilin-1, VEGF-A_165_, VEGF-A_165_/NRP-1 complex, protein-ligand interaction, peptide ligands

## Abstract

The structure-activity relationship of branched H-Lys(*h*Arg)-Dab-Dhp-Arg-OH sequence analogues, modified with Cys-Asp or Cys at N-terminal amino acids (Lys, *h*Arg), in VEGF-A_165_/Neuropilin-1 complex inhibition is presented. The addition of Cys residue led to a 100-fold decrease in the IC_50_ value, compared to the parent peptide. The change occurred regardless of coupling Cys to the free N-terminal amino group present in the main or the side chain. A few analogues extended by the attachment of Cys at the N-terminus of several potent NRP-1 peptide ligands documented in the literature are also presented. In all studied cases, the enhancement of inhibitory properties after the addition of Cys at the N-terminus is observed. It is particularly evident for the tetrapeptide derived from the C-terminus of VEGF-A_165_ (KPRR), suggesting that extending the K/RXXK/R motif (CendR) with the Cys moiety can significantly improve affinity to NRP-1 of CendR peptides.

## 1. Introduction

Neuropilin-1 (NRP-1) is a cell surface receptor involved in the development of axon guidance and also in physiological, as well as pathological, angiogenesis processes, including cancer [1,2,3]. NRP-1, as a co-receptor for class 3 semaphorin and multiple growth factors, including epidermal growth factor (EGF), vascular endothelial growth factor (VEGF), PDGF, platelet-derived growth factor (PlGF), hepatocyte growth factor (HGF), and transforming growth factor β (TGF-β), cooperatively enhances the activity of the respective receptor tyrosine kinases (RTKs) in cell signaling systems [4]. Vascular endothelial growth factor A_165_ (VEGF-A_165_) is considered to be the most significant modulator of angiogenesis [5,6,7], and NRP-1 enhances its binding to VEGFR-2 [8,9]. There is evidence that NRP-1, despite the lack of catalytic activity, also serves as an independent mediator of blood vessel development in cancerous tumors and that its expression on the surface of cancerous cells is increased and often associated with poor prognosis [10,11,12,13]. These results indicate that searching for new NRP-1 inhibitors of pathological angiogenesis, including functional peptides, could play a crucial role in drug development (e.g., in oncology).

The VEGF-A_165_ fragment encoded by exons 7 and 8 is mainly responsible for protein interaction with NRP-1 [14,15,16,17]. The binding pocket of the NRP-1 b1 domain is shallow, and only the C-terminal Arg of the ligand is able to directly enter into it [6,18]. Screening of phage libraries led to the identification of peptides possessing a R/KXXR/K consensus motif embedded in their terminal sequence, which showed a high binding affinity to NRP-1 [18]. As with VEGF-A_165_, the C-terminal Arg (or rarely, Lys) was important for NRP-1-binding. Blocking the free carboxyl group of this Arg residue by amidation or adding another amino acid eliminated ligand binding to the receptor. The strict requirements of the R/KXXR/K C-terminal structural motif in NRP-1 peptidic ligands is called the C-end rule (CendR) [19]. The CendR fragment is present in the structure of active peptidic NRP-1 ligands described in the literature, e.g., heptapeptide A7R (ATWLPPR) [19,20,21], shorter analogues of A7R [22], tuftsin [23], and penetrating iRGD peptide [24] as well as the C-terminus of VEGF-A_165_ (CDKPRR) encoded by the exon 8 and multiple viruses’ capsid proteins, e.g., human T-cell lymphotropic virus type 1 (HTLV-1) [25,26] and Epstein–Barr virus (EBV) [27].

Another characteristic feature of the VEGF-A_165_ structure is the presence of eight invariant cysteine (Cys) residues associated with intermolecular and intramolecular disulfide bonds [28]. The N-terminal fragment of the VEGF-A_165_ domain ERTCDKPRR encoded by exons 7 and 8, as well as its shorter analogue CDKPRR, showed nearly 100% inhibition of the formation of the VEGF-A_165_/NRP-1 complex at a concentration of 100 μM [29]. The replacement of Cys with Ser at positions 4 and 6 of the former peptide and deletion of Cys in the latter sequence caused a significant decrease in the affinity for NRP-1. These results suggested that Cys may have a key role, adjacent to the CendR motif, in NRP-1 interaction. Various types of ligand-receptor interactions involving the thiol group of Cys residues are described in the literature. Experimental and theoretical evidences indicate that Cys can be a hydrogen bond donor and/or acceptor, where its hydrogen-bonding interactions influence reactivity and, thus, biological functions [30,31]. The strength of the hydrogen bond to the Cys thiol/thiolate (-SH/-S^−^) depends also on its hydrogen-bonding partner and its local environment. There is also a clear tendency of the sulphur atom to interact with the surface of the aromatic ring (S-π interaction) [30,32,33,34]. Thiol–disulfide exchange reactions between ligand-containing Cys and the protein may also occur [35,36]. All of these interactions may play a crucial role in the formation of the ligand-receptor complex.

Among the antiangiogenic molecules, a groundbreaking discovery was the A7R heptapeptide, isolated using phage display library screening, which in vivo decreases breast cancer angiogenesis and growth [19,20,21]. Based on the A7R peptide, which showed the ability to inhibit VEGF-A_165_/NRP-1 complex formation with a high IC_50_ value (IC_50_ = 80 μM) [21], and respecting CendR rules, we previously obtained short-branched pentapeptides, with a general sequence Lys(*h*Arg)-AA^2^-AA^3^-Arg, that have reduced IC_50_ values (IC_50_ = 2–20 μM) [37,38]. In the present study, we decided to examine how an additional Cys-Asp (CD) fragment (present at the N-terminus of the C-terminal hexapeptide of VEGF-A_165_–CDKPRR) or Cys (C) residue will influence affinity for NRP-1 of the best analogue of our branched peptides, namely Lys(*h*Arg)-Dap-Dhp-Arg. Following this strategy, four analogues were designed and synthesized. The parent branched peptide has two free amino groups at N-terminus: one in the main chain (α-amino group of Lys) and the second one in the branched part of the molecule (α-amino group of hArg). Thus, we designed analogues in which additional Cys-Asp or Cys were placed in both positions. Enzymatic stability studies were performed for the most promising branched peptides. Moreover, to further evaluate the effect of the cysteine addition, linear peptidic ligands based on active compounds described in the literature, with additional Cys on the N-terminus, were synthesized and tested for affinity to NRP-1.

## 2. Materials and Methods

### 2.1. Materials

Unless otherwise specified, reagents were obtained from commercial sources and used without further purification. Fmoc-Arg(Pbf) Wang resin was purchased from Activotec (Cambridge, UK). Amino acids and coupling reagents were obtained from Iris Biotech (Marktredwitz, Germany). Solvents and reagents were purchased from Merck (Darmstadt, Germany). Recombinant human NRP-1 and biotinylated human VEGF-A165 were purchased from R&D Systems (Minneapolis, MN, USA). Chemiluminescent substrate was purchased from Thermo Scientific (Waltham, MA, USA). Luminescence was measured using a Tecan Infinite F200Pro microplate reader (Männedorf, Switzerland).

### 2.2. Peptides Synthesis

The synthesis of all peptides was carried out manually on the preloaded Fmoc-Arg(Pbf) Wang resin with a capacity of 0.39 mmol/g (0.5 g) following the Fmoc chemistry. Coupling of 2 eq amino acids (0.4 mmol) was done using 2 eq HATU (0.4 mmol) and 5 eq DIPEA (1 mmol) in DMF (5 mL). Completion of coupling was checked using a Kaiser or chloranil test. Fmoc deprotection step was done using 20% piperidine in DMF. Tetrakis(triphenylphosphine)palladium(0) (0.016 mmol; 0.1 eq) in the presence of 20 eq PhSiH_3_ (3.2 mmol) in DCM was used for the Alloc deprotection step. Final compounds were cleaved from the resin with the use of 5 mL TFA:DTT:H_2_O:TIS (88:5:5:2, *v*:*w*:*v*:*v*) for 3 h and then precipitated by a dropwise addition into a cold diethyl ether. Synthesized compounds were purified on a Shimadzu Prominence preparative HPLC system (Duisburg, Germany) equipped with a Phenomenex Jupiter Proteo C12 90 AXIA Å 10 µm 250 × 21.2 mm column (Torrance, CA, USA). Pure compounds were analyzed with the Shimadzu Prominence analytical HPLC system (Duisburg, Germany) and Sciex 6600TOF mass spectrometer (Concord, Ontario, Canada). Detailed analytical method description and data for purified compounds (HPLC-UV, HRMS and HRMS/MS) are available in the Supplementary Materials.

### 2.3. Competitive NRP-1-Binding Assay

The method used was similar to the one previously described [29,38,39,40,41]. The first step was a coating of a 96-well plate using 100 μL (200 ng/well) of recombinant human NRP-1. After overnight incubation at 4 °C, nonspecific binding was blocked with 0.5% BSA in PBS. Next, 50 µL of peptide in PBS in selected concentrations and 50 µL of human (bt)-VEGF-A_165_ (400 ng/mL) in PBS containing 4 µg/mL of heparin were added and incubated for 2 h at r.t. After washing, wells were treated with streptavidin-horseradish peroxidase conjugate in PBS (1:8000) for 45 min. Final washing was followed by an addition of 100 µL chemiluminescent substrate and the immediate quantitative determination of the luminescence. Wells incubated with (bt)-VEGF-A_165_ only served as a positive control (P), while not-coated by NRP-1 wells were used for a negative control (NS). Percentages of inhibition were calculated by the following formula:

100% − [[(S − NS)/(P − NS)]·100%],(1)

where S is the signal intensity obtained from wells incubated with the peptide. Presented data are the mean ± SEM of two or three independent experiments, each performed in triplicate.

### 2.4. Degradation Assay in Human Plasma

This assay was performed according to the method described previously [38,41]. First, human blood plasma was preactivated in an Eppendorf ThermoMixer^®^ Comfort (Hamburg, Germany) for 20 min at 37 °C (350 rpm). Next, the peptide dissolved in water (6 mg/mL; ~5 μmol/mL) was mixed with human plasma (~1 μmol/mL final concentration) and incubated in the same conditions. One-hundred microliters of the mixture was collected in 1.5-mL microtubes at selected time intervals (0–96 h), followed by protein precipitation by adding 100 μL of 98% ethanol and by vortexing for 1 min (3000 min^−1^). Samples were centrifuged for 10 min at 4 °C (11,000 *g*), and 100 μL of supernatant was collected and lyophilized. Reconstituted in 100 μL of water, samples were subjected to RP-HPLC analysis. LC-MS analyses with a Shimadzu LCMS-2020 mass spectrometer (Duisburg, Germany) were done for selected samples. A detailed description of the method can be found in the Supplementary Materials. All results are represented as an average, with error bars indicating ± SEM determined from the results of two independent experiments performed in duplicates.

### 2.5. Statistical Analysis

IC_50_ of the peptides were calculated using the nonlinear regression function with Prism (GraphPad Software, San Diego, CA, USA). The same software was used to calculate and present the plasma stability assay results. All results are represented as an average, with error bars indicating ± SEM.

### 2.6. Ethics

The study was conducted in full accordance with ethical principles in accordance with the ICH E6 (R2) Guideline for Good Clinical Practice and the Code of Good Customs in Science developed by the Polish Academy of Sciences. The Rector’s Commission for Ethics of Scientific Research with Human Participation, University of Warsaw, Warsaw, Poland gave the ethical approval (approval no. 51/2020). A signed consent for using plasma was obtained from volunteers.

## 3. Results and Discussion

### 3.1. Peptides Synthesis

**Scheme 1:**
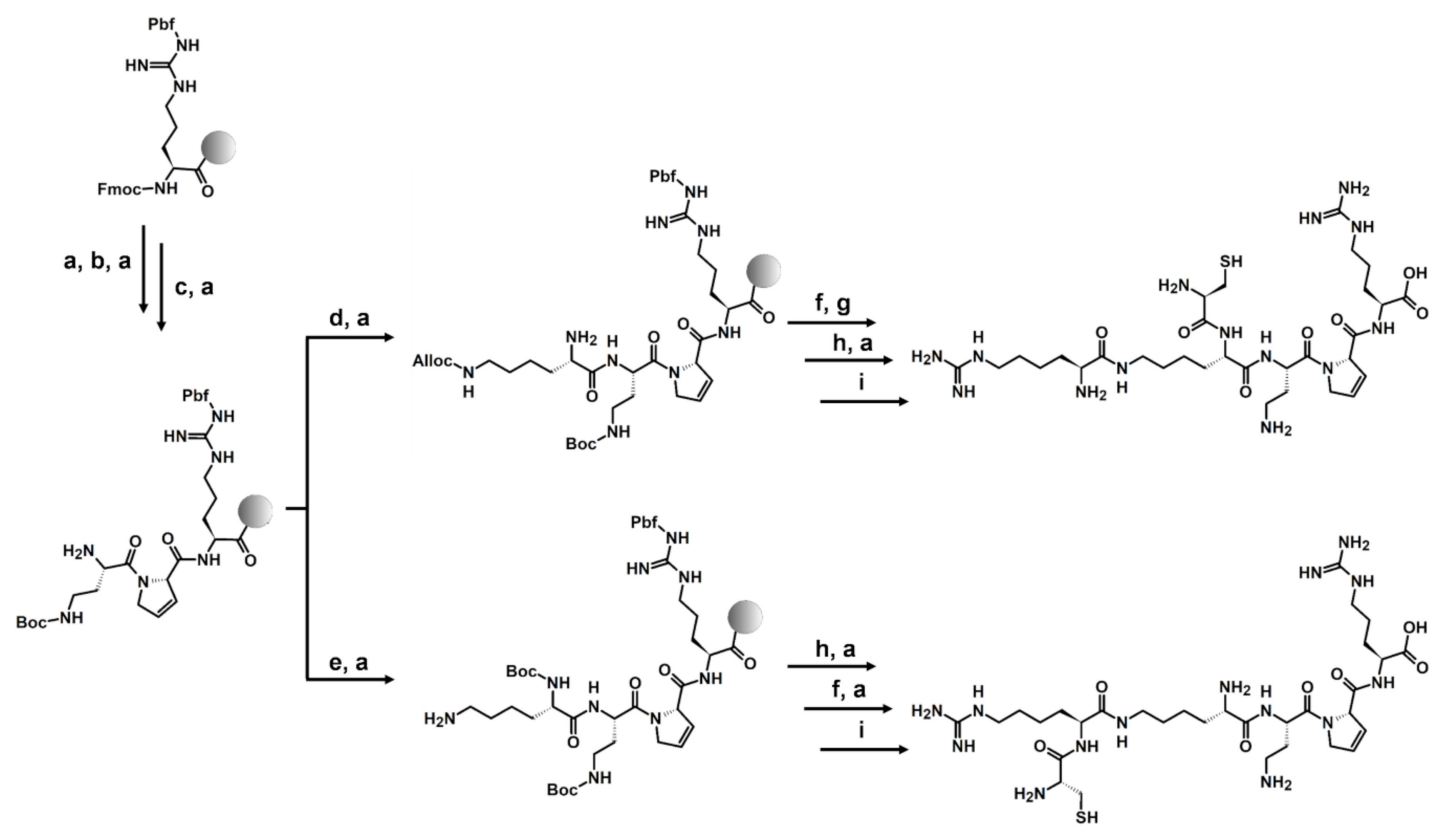
The solid-phase peptide synthesis (SPPS) strategy on the example of peptides **3** and **4**: (**a**) 20% piperidine in DMF; (**b**) 2 eq Fmoc-Dhp-OH, HATU, and DIPEA; (**c**) 2 eq Fmoc-Dab(Boc)-OH, HATU, and DIPEA; (**d**) 2 eq Fmoc-Lys(Alloc)-OH, HATU, and DIPEA; (**e**) 2 eq Boc-Lys(Fmoc), HATU, and DIPEA; (**f**) 2 eq Fmoc-Cys(Trt)-OH, HATU, and DIPEA; (**g**) Pd(PPh_3_)_4_ and PhSiH_3_; (**h**) 2 eq Fmoc-*h*Arg(Pbf)-OH, HATU, and DIPEA; and (**i**) TFA:DTT:H_2_O:TIS.

The synthesis of compounds was carried out on the preloaded Fmoc-Arg(Pbf)-Wang resin following the Fmoc chemistry (Scheme 1) [42]. The standard coupling HATU/DIPEA protocol was used [43]. The obtained compounds were cleaved from the resin using the TFA:DTT:H_2_O:TIS mixture, purified, and analyzed using RP-HPLC, HRMS, and HRMS/MS.

### 3.2. VEGF-A_165_/NRP-1 Complex Inhibition Assay

Peptides were tested by the bt-VEGF-A_165_/NRP-1 complex inhibition assay with chemiluminescent detection, as previously described [29,38,39,40,41]. Assays of peptides **1** and **2**, with additional Cys-Asp residues, have been carried out at concentrations between 0.1 μM and 50 μM. The obtained results show a slight increase in the affinity of these analogues for the receptor relative to the parent sequence Lys(*h*Arg)-Dab-Dhp-Arg (IC_50_ = 4.3 μM) (Table 1). For both compounds, the calculated IC_50_ value is two times lower, regardless of whether the Cys-Asp fragment was attached to the N-terminus (IC_50_ = 2.5 μM) or to the *h*Arg (IC_50_ = 2.0 μM) (Table 1). Extending the sequence by a Cys-Asp fragment did not significantly reduce the IC_50_, but neither did it negatively affect the inhibitory properties of the compounds. It was therefore tested how the addition of only the Cys residue would affect the affinity of the parent peptide (PP) for the NRP-1 receptor. The amino acid was inserted in the same places as the Cys-Asp fragment previously. Compounds **3** and **4** were tested by the bt-VEGF-A_165_/NRP-1 inhibition assay at 1–50 μM concentrations. However, results in this area did not allow the calculation of IC_50_. Only fluctuations in inhibition values for the given concentrations were observed within ~80%, which occurs in the case of a range of concentrations in the flat area of the curve (plateau) describing the concentration-reaction relationship.

We next adjusted the concentration range for compounds **3** and **4** and set it between 0.005–1 μM. The IC_50_ values obtained for both analogues, 80 nM for compound **3** and 60 nM for **4**, were two orders of magnitude lower than the PP. The small difference in affinity for NRP-1 between these compounds shows that the Cys residue has an extremely beneficial effect on their inhibitory ability, regardless of the selected amino acid positions.

**Figure 1:**
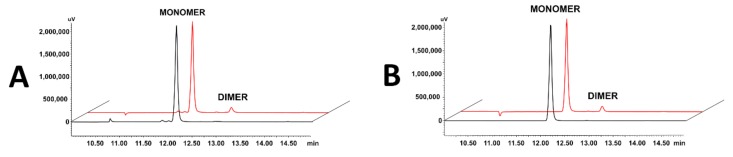
Superimposed chromatograms of peptides **3** (**A**) and **4** (**B**) incubated in PBS at 22 °C for 0 h (black) and 4 h (red).

Peptides containing a single Cys residue can form dimers by the oxidation of the cysteine side chain thiols, linking two chains together by a disulphide bridge. Disulphide bond formations may occur by air oxidation in a diluted aqueous solution of the peptide at a neutral pH. To confirm that dimers were not formed during affinity assays, compounds **3** and **4** were incubated in PBS at 22 °C for 4 h to reproduce conditions during the ELISA-like test, and then samples were tested by RP-HPLC with UV detection. The results of these experiments showed that the dimerization of the compounds occurs minimally, at the level of 5% of the total concentration (peak area of dimer/monomer ratio); therefore, the obtained IC_50_ values were considered as results for the monomers (Figure 1).

Due to the positive results of Cys introduction into the parent-branched Lys(*h*Arg)-Dab-Dhp-Arg peptide, new analogues, based on NRP-1 peptidic ligands known from the literature and with linear structures, were synthesized to test if an increase of their affinity for NRP-1 will also appear for a nonbranched sequence (Table 2). Designed compounds were modified by an N-terminal addition of Cys to known tetrapeptides or by shortening the sequence of longer peptides to preserve the Cys-X-X-X-Arg motif. The first modified compound was the A7R peptide with an ATWLPPR sequence, which has been previously identified by the phage display technique and was shown to bind specifically to NRP-1 (IC_50_ = 60–84 µM) [19,21,38]. This binding strongly inhibits the activation of NRP-1 by VEGF-A_165_, and the LPPR sequence is responsible for 75% of this inhibition [44]. Since the LPPR peptide sequence is essential for the inhibition of VEGF activity, Cys on the N-terminus was added to this peptide fragment to preserve the Cys-X-X-X-Arg motif. Competitive assay results for peptide **5** show that sequence-shortening to the N-terminal tetrapeptide and the addition of Cys decreased IC_50_ seven-fold compared to the A7R peptide. Next, tuftsin (TKPR), which is an endogenous peptide that mimics the C-terminal tail of VEGF-A_165_ and selectively binds to NRP-1 [23], was modified. Our data from the initial tests show that tuftsin’s IC_50_ value is 100 µM, and this result coincides with the literature data [23]. This peptide is important due to a breakthrough in research on NRP-1, which was its co-crystallization with b1b2 subdomains’ NRP-1 [45]. The results determined that a key fragment in the ligand-receptor interaction is the C-terminal Arg guanidine group. In this case, additional Cys on the N-terminus (peptide **6**) increased ligand affinity more than 11-fold. The last tested compound was peptide **7**, based on the key fragment for VEGF-A_165_/NRP-1 interaction, the CDKPRR sequence encoded by exon 8 [29,46]. As we previously mentioned, the CDKPRR peptide blocked this interaction with percentages of inhibition of almost 100% at 100 µM, but the lack of Cys at the N-terminal (DKPRR) causes great deterioration of receptor affinity (~20% inhibition at 100 µM) [29,41]. Compound **7** shows inhibition of the VEGF-A_165_/NRP-1 complex formation with IC_50_ = 190 nM, which is an extraordinary improvement of its affinity (Table 2). Overall, the addition of Cys at the N-terminus enhances the affinity of tested peptides, showing that the cysteine rule (CysR) may be an important factor in optimizing active sequences.

### 3.3. Enzymatic Stability in Human Plasma

In addition to a high receptor affinity, enzyme resistance also plays an important role in the design of new biologically active compounds. Therefore, we performed in vitro enzymatic stability studies of peptides **3** and **4** in human blood plasma, to investigate whether the introduced Cys affected the half-life of the new compounds. Most peptidases, including those present in plasma, have certain preferences for cleavage regions. Many enzymes require a basic amino acid at the P1 position or small amino acids (Ala, Cys, Gly, and Ser) at the P1 and P1’ positions for their action [47].

**Figure 2:**
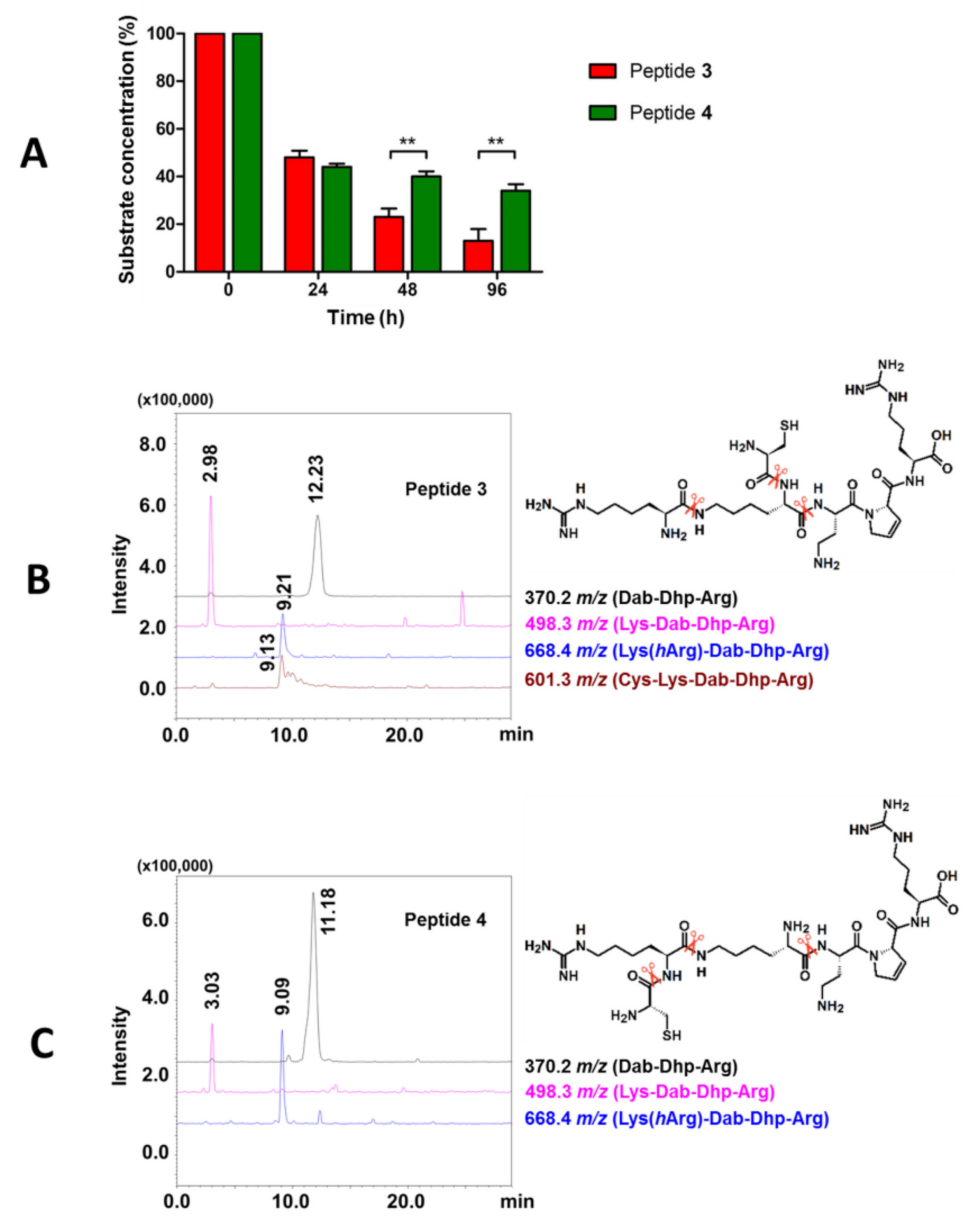
(**A**) Comparison of the in vitro metabolic stability of peptides **3** and **4**. Results are presented as the mean ± SEM of two independent experiments performed in duplicates. Differences between peptides concentrations at various time intervals were analyzed by a two-way ANOVA with Bonferroni’s post-tests (** *p* < 0.01). Extracted ion chromatograms (XICs) of detected metabolites and potential bonds cleaved by enzymes (scissors represent identified proteases cleavage sites) of (**B**) peptide **3** and (**C**) peptide **4**.

Compounds were incubated in human plasma at 37 °C, and at selected time intervals, samples were analyzed using RP-HPLC and LC-MS, as described previously [38,43]. Figure 2A shows the concentration decline of each of the tested peptides during plasma incubation. After 24 h, almost 50% of each compound’s initial concentration was still present in the plasma. Following the next time intervals, we observed a significant degradation of peptide **3** (~20% after 96 h) but a much slower decrease in the concentration of peptide **4** (~40% after 96 h). This is probably due to the slower detachment of Cys residue from the side chain (peptide **4**) compared to detachment from the main chain (peptide **3**). Presented data show that additional Cys at the N-terminus of the sequence reduced the enzymatic stability of the peptide compared to the parent peptide with t_1/2_ = 51 h [38]. However, unmodified peptides tend to have short half-lives counted rather in minutes, e.g., ghrelin [48] and endomorphins [49], and modifications lead to extending this time often to only a few hours [49,50,51]. Thus, our results show that compounds **3** and **4** are relatively resistant to proteolytic enzymes.

Identification of the metabolites was hampered by reactions occurring during incubation. Substrates and fragments with the thiol group could form disulphide bonds in various combinations; nevertheless, we determined, at least in part, which amide bonds undergo proteolysis. Enzymatic cleavage of the Cys-Lys/Cys-*h*Arg, Lys-ε-*h*Arg, and Lys-Dab bonds were found (Figure 2B,C). Obtained RP-HPLC results show that, for both compounds, Cys is the first amino acid that is cleaved by enzymes (see Figures S9 and S11 in the Supplementary Materials). However, the site of attachment of the Cys residue seems to affect the resistance of the peptide. We hypothesize that compound **4** may have increased enzymatic resistance due to the Cys and *h*Arg amide bond’s limited accessibility for an enzymatic cleave.

## 4. Conclusions

In this study, we have synthesized a new group of analogues based on our previously described branched H-Lys(*h*Arg)-Dab-Dhp-Arg-OH sequence, which is a potent ligand of the NRP-1 receptor. We have now extended this sequence at the N-terminus with a Cys-Asp or Cys fragment. The addition of the Cys-Asp fragment, either at the N-terminus of the main or branched chain (α-amino group of Lys or α-amino group of the *h*Arg), decreased the affinity of the peptidomimetics to NRP-1 by approximately two-fold (peptides **1** and **2**). However, elongation of the parent peptide by the Cys residue alone resulted in a significant 50- and 70-fold decrease of the IC_50_ values for peptides **3** and **4,** respectively. This significant increase in the inhibitory effect occurred regardless of the place of the Cys attachment at the N-terminus. We also performed the enzymatic stability study of our best analogues, which demonstrated that our analogues are relatively stable in human plasma compared to natural active peptides, especially an analogue with Cys attached to the side chain (coupled with *h*Arg).

Furthermore, we synthesized extended (Cys) analogues of several potent NRP-1 ligands documented in the literature. In all our experiments, we observed the enhancement of inhibitory properties after the addition of Cys. It was particularly evident for the peptide derived from the C-terminus of VEGF-A_165_ (DKPRR), suggesting that extending the K/RXXK/R motif (CendR) with the Cys moiety can significantly improve the affinity to NRP-1 of the CendR peptides. The increase in the inhibitory properties of the peptides with Cys added at the N-terminus may be due to the possibility of additional interactions with NRP-1 through the SH group (hydrogen bond formation or ionic interaction). Thus, we assume that such observed enhancements of the inhibitory activity after the addition of Cys could be a more general rule, named in this report as CysR, which may complement the CendR rule, but the role of Cys in ligand-protein interactions still requires thorough investigation. The presented peptides have potential applications in drug design, but further studies should be performed. The next step will be the determination of the thiol group’s importance in the ligand/NRP-1 interaction and optimization of the sulphur-containing side chain length. An important part of the research will be in vitro experiments which allow to prove that ligands bind to NRP-1 on cells’ surfaces.

## Figures and Tables

**Table 1:** Inhibitory effect of branched peptides **1**–**4** on the VEGF-A_165_/NRP-1 complex formation.

Compound	Sequence	logIC_50_	IC_50_ (µM)
**PP ^1^**	H-Lys(*h*Arg)-Dab-Dhp-Arg-OH	−5.54 ± 0.04	4.3 ^2^
**1**	H-Cys-Asp-Lys(*h*Arg)-Dab-Dhp-Arg-OH	−5.60 ± 0.03	2.5
**2**	H-Lys(Cys-Asp-*h*Arg)-Dab-Dhp-Arg-OH	−5.70 ± 0.05	2.0
**3**	H-Cys-Lys(*h*Arg)-Dab-Dhp-Arg-OH	−7.079 ± 0.1316	0.08
**4**	H-Lys(Cys-*h*Arg)-Dab-Dhp-Arg-OH	−7.194 ± 0.0939	0.06

^1^ PP, parent peptide. ^2^ Data already published [38]. Unnatural amino acid abbreviations: Dab, 2,4-diaminobutyric acid and Dhp, 3,4-dehydroproline. Results are presented as the mean ± SEM of two (peptides **1** and **2**) or three independent experiments, each performed in triplicate.

**Table 2:** Inhibitory effects of known peptidic ligands from the literature, modified by a Cys addition at the C-terminus, on the VEGF-A_165_/NRP-1 complex formation.

Compound	Sequence	logIC_50_	IC_50_ (µM)	Reference Peptide (RP)	RP IC_50_ (µM)
**5**	CLPPR	–4.94 ± 0.08	11.4	A7R ^1^	80 (19,38)
**6**	CTKPR	–5.03 ± 0.08	9.3	Tuftsin ^2^	≥100 (23) ^3^
**7**	CKPRR	–6.727 ± 0.762	0.19	DKPRR	>>100 (29,41)

^1^ A7R sequence: ATWLPPR. ^2^ Tuftsin sequence: TKPR. *^3^* Obtained percentage inhibitory effect of tuftsin for the 100 µM concentration was 46% ± 3%. Results are presented as the mean ± SEM of two (peptides **5** and **6**) or three independent experiments, each performed in triplicate.

## Supplementary Materials

The following are available online at https://www.mdpi.com/2218-273X/10/3/448/s1, Figures S1 and S2: HPLC-UV chromatogram of peptides 1–2 at 190 nm, HRMS spectrum, and MS/MS fragmentation of [M+2H]^2+^, Figures S3–S7: HPLC chromatogram of peptides 3–7 at 190 nm, HRMS spectrum, and MS/MS fragmentation of [M+H], Figure S8: Dose-response curves of peptides 1–7, Figure S9: Full chromatogram of peptide 3 after degradation in different time intervals, Figure S10: Zoom-in peptide 3 signal after degradation in different time intervals, Figure S11: Full chromatogram of peptide 4 after degradation in different time intervals, Figure S12: Zoom-in peptide 4 signal after degradation in different time intervals, Table S1: Molecular weight, reaction yields, and HPLC analytical data of compounds 1–7, Table S2: HRMS analytical data of compounds 1–7, Table S3: MS/MS analytical data of compounds 1–7.
