# Supplementary material for: Does Cysteine Rule (CysR) Complete the CendR Principle? Increase in Affinity of Peptide Ligands for NRP-1 Through the Presence of N-Terminal Cysteine
Source: Biomolecules. 2020 Mar 13;10(3):448. doi: 10.3390/biom10030448 (PMC7175122; doi:10.3390/biom10030448)
Supplement: Supplementary file 1 [file biomolecules-10-00448-s001.pdf]

SUPPLEMENTARY MATERIALS

Does Cysteine rule (CysR) complete the CendR principle? Increase in affinity of peptide ligands for NRP-1 through the presence of N-terminal cysteine

Anna K. Puszko [1], Piotr Sosnowski [2], Françoise Raynaud [3,4,5], Olivier Hermine [3,4,5], Gérard Hopfgartner [2], Yves Lepelletier [3,4,5,*], Aleksandra Misicka [1,6,*]

[1] *Faculty of Chemistry, University of Warsaw, Pasteura 1, 02-093 Warsaw, Poland*

[2] *Department of Inorganic and Analytical Chemistry, University of Geneva, 24 Quai Ernest Ansermet, CH-1211 Geneva 4, Switzerland*

[3] *Université de Paris, Imagine Institute, 24 boulevard Montparnasse, 75015 Paris, France*

[4] *INSERM UMR 1163, Laboratory of cellular and molecular basis of normal hematopoiesis and hematological disorders: therapeutical implications, 24 boulevard Montparnasse,75015 Paris, France*

[5] *CNRS ERL 8254, 24 boulevard Montparnasse, 75015 Paris, France*

[6] *Department of Neuropeptides, Mossakowski Medical Research Centre, Polish Academy of Sciences, Pawinskiego 5, 02-106 Warsaw, Poland*

[*] Correspondence: misicka@chem.uw.edu.pl (A.M.), y.lepelletier@gmail.com (Y.L.)

Table of contents

1. Analytical data of synthesized peptides **2**

2. Dose-response curves of synthesized peptides **13**

3. Analytical data of serum degradation **14**

1. Analytical data of synthesized peptides

Purity of compounds (> 98%) was determined using RP-HPLC. Analysis of pure products was carried out by HPLC with a Prominence HPLC system (binary pump system LC-20AD and autosampler SIL-20AC HT coupled to an SPD-20A UV detector). Chromatographic separation was achieved on Phenomenex Jupiter Proteo C12 column 90Å 4 µm 250 × 4.6 mm at 35˚C. Elution was performed with a gradient as follows:

Method 1: 0–3 min 0%; 3–23 min 18% Mobile phases consisted of H_2O:TFA (99.95:0.05 v/v, phase A) and ACN:TFA (99.95:0.05 v/v, phase B) at a flow rate of 1.2 mL/min.

Method 2: 0–25 min 25% Mobile phases consisted of H_2O:TFA (99.95:0.1 v/v, phase A) and ACN:TFA (99.95:0.1 v/v, phase B) at a flow rate of 1 mL/min.

Method 3: 0–20 min 20% Mobile phases consisted of H_2O:TFA (99.95:0.1 v/v, phase A) and ACN:TFA (99.95:0.1 v/v, phase B) at a flow rate of 1 mL/min.

UV spectra were recorded at 190 nm. Purity of compounds was estimated using the peak areas. High-resolution mass spectra (HRMS) and high-resolution fragmentation spectra (MS/MS) were recorded on a SCIEX 6600TOF instrument with an ESI ionization source and by infusion at 10 µl/min. Solutions (0.1 mg/ml) of each compound were prepared in 50% MeOH 0.1% FA. The resolution power was of about 30,000 at *m/z* 300. The mass reported is containing the most abundant isotopes with a mass error < 10 ppm. The electrospray ionization (ESI) was operated in positive mode. Curtain gas (CUR) was set to 25 psi. Nebulizing gas (GS1) was set to 20 psi, and drying gas (GS2) was set to 15 psi. Needle voltage (ISVF) was set to 5kV, and temperature (TEM) was set to 50 ºC. Declustering potential (DP) was set to 80 V. To induce fragmentation, collision energy voltage (CE) was set to 30 V, and collision energy spread voltage (CES) was set to 15 V. Mass spectrometer was operated in TOFMS and (MS/MS) modes in a range adjusted to the analyte's predicted mass.

Theoretical $(M+nH)^{n+}$ values and errors were calculated using the Mass Calculators tool integrated with the spectrometer operating software.

Table S1. Molecular weight, reaction yields, and HPLC analytical data of compounds **1**-**7**.

Compound	MW (g/mol)	MW_{+TFA} (g/mol)	Yield	RT (min)
**1**	886.03	1456.03	41%	11.03[a]
**2**	886.03	1456.03	46%	10.87[a]
**3**	770.95	1340.95	51%	10.73[a]
**4**	770.95	1340.95	48%	10.50[a]
**5**	584.73	812.73	65%	19.55[b]
**6**	603.74	945.74	68%	12.90[c]
**7**	658.81	1114.81	68%	13.21[c]

[a] Compound was analyzed using method 1; [b] Compound was analyzed using method 2; and [c] Compound was analyzed using method 3.

Table S2. HRMS analytical data of compounds **1-7**.

Compound	Molecular formula	$(M+H)^+$ calculated	$(M+H)^+$ found	Error (ppm)	$(M+2H)^{2+}$ calculated	$(M+2H)^{2+}$ found	Error (ppm)	$(M+3H)^{3+}$ calculated	$(M+3H)^{3+}$ found	Error (ppm)
**1**	$C_{35}H_{63}N_{15}O_{10}S$	886.4676	886.4715	4.4	443.7374	443.7389	1.5	296.1607	296.1613	2.0
**2**	$C_{35}H_{63}N_{15}O_{10}S$	886.4676	886.4708	3.6	443.7374	443.7388	3.2	296.1607	296.1607	0.3
**3**	$C_{31}H_{58}N_{14}O_7S$	771.4406	771.4396	-1.3	386.2240	386.2245	1.3	257.8184	257.8194	3.9
**4**	$C_{31}H_{58}N_{14}O_7S$	771.4406	771.4402	-0.6	386.2240	386.2250	2.6	257.8184	257.8199	5.8
**5**	$C_{25}H_{44}N_8O_6S$	585.3177	585.3192	2.6	293.1625	293.1634	3.1	-	-	-
**6**	$C_{24}H_{45}N_9O_7S$	604.3235	604.3248	2.2	302.6654	302.6669	5.0	-	-	-
**7**	$C_{26}H_{50}N_{12}O_6S$	659.3779	659.3793	3.5	330.1921	330.1941	6.1	220.4638	220.4652	6.4

Table S3. MS/MS analytical data of compounds **1**-**7**.

Peptide 1			
**fragment & formula**	***m/z* calculated**	***m/z* found**	**Error (ppm)**
y_1 $C_6H_{15}N_4O_2^+$	175.1190	175.1178	-6.9
y_2 - NH_3 $C_{11}H_{17}N_4O_3^+$	253.1280	253.1295	5.9
y_2 $C_{11}H_{20}N_5O_3^+$	270.1561	270.1552	-3.3
y_4^{2+} $C_{28}H_{55}N_{13}O_6^+$	334.7194	334.7179	-4.5
Lys(*h*Arg)Dab $C_{17}H_{35}N_8O_3^+$	399.2827	399.2814	-3.3
$[M+2H-NH_3]^{2+}$ $C_{35}H_{62}N_{14}O_{10}S^+$	435.2242	435.2228	-3.2
b_4 $C_{24}H_{45}N_{10}O_7S^+$	617.3188	617.3184	-0.6

Peptide 2			
**fragment & formula**	***m/z* calculated**	***m/z* found**	**Error (ppm)**
y_1 $C_6H_{15}N_4O_2^+$	175.1190	175.1193	1.7
y_2 - NH_3 $C_{11}H_{17}N_4O_3^+$	253.1280	253.1294	5.5
y_2 $C_{11}H_{20}N_5O_3^+$	270.1561	270.1564	1.1
y_4^{2+} $C_{28}H_{55}N_{13}O_6^+$	334.7194	334.7190	-1.2
CysAsp*h*Arg $C_{14}H_{25}N_6O_5^+$	389.1602	389.1594	-2.1
Lys(*h*Arg)Dab $C_{17}H_{35}N_8O_3^+$	339.2827	339.2820	-1.8
LysDabΔProArg $C_{21}H_{40}N_9O_5^+$	498.3147	498.3134	-2.6
b_4 $C_{24}H_{45}N_{10}O_7S^+$	617.3188	617.3182	-1.0

Peptide 3			
**fragment & formula**	***m/z* calculated**	***m/z* found**	**Error (ppm)**
y_1 $C_6H_{15}N_4O_2^+$	175.1190	175.1175	-8.6
y_2 $C_{11}H_{20}N_5O_3^+$	270.1561	270.1551	-3.7
b_2 $C_{16}H_{32}N_7O_3S^+$	402.2282	402.2282	-0.7
b_3-NH_3 $C_{20}H_{37}N_8O_4S^+$	485.2653	485.2626	-5.6
b_3 $C_{20}H_{40}N_9O_4S^+$	502.2919	502.2896	-4.6
b_4 $C_{25}H_{45}N_{10}O_5S^+$	597.3290	597.3270	-3.3

Peptide 4			
**fragment & formula**	***m/z* calculated**	***m/z* found**	**Error (ppm)**
y_1 $C_6H_{15}N_4O_2^+$	175.1190	175.1176	-8.0
LysDab $C_{10}H_{21}N_4O_2^+$	229.1659	229.1656	-1.3
Cys*h*Arg - NH_3 $C_{10}H_{17}N_4O_2S^+$	257.1067	257.1058	-3.5
y_2 $C_{11}H_{20}N_5O_3^+$	270.1561	270.1551	-3.7
b_2-NH_3 $C_{20}H_{37}N_8O_4S^+$	485.2653	485.2647	-1.2
b_2 $C_{20}H_{40}N_9O_4S^+$	502.2919	502.2921	0.4

Peptide 5			
**fragment & formula**	***m/z* calculated**	**$(M+H)^+$ found**	**Error (ppm)**
y_1 $C_6H_{15}N_4O_2^+$	175.1190	175.1195	2.9
b_2 $C_9H_{17}N_2O_2S^+$	217.1005	217.1013	3.7
y_2 - NH_3 $C_{11}H_{19}N_4O_3^+$	255.1452	255.1462	3.9
y_2 $C_{11}H_{22}N_5O_3^+$	272.1717	272.1729	4.4
b_3 $C_{14}H_{24}N_3O_3S^+$	314.1533	314.2544	3.5
y_3- NH_3 $C_{16}H_{26}N_5O_4^+$	352.1979	352.1996	4.8
y_3 $C_{16}H_{29}N_6O_4^+$	369.2245	369.2257	3.3
b_4 $C_{19}H_{31}N_4O_4S^+$	411.2061	411.2068	1.7
y_4 $C_{22}H_{40}N_7O^+$	482.3085	482.3095	2.1

Peptide 6			
**fragment & formula**	***m/z* calculated**	***m/z* found**	**Error (ppm)**
y_1 $C_6H_{15}N_4O_2^+$	175.1190	175.1193	1.7
y_2 - NH_3 $C_{11}H_{19}N_4O_3^+$	255.1452	255.1459	2.7
y_2 $C_{11}H_{22}N_5O_3^+$	272.1717	272.1726	3.3
b_3 $C_{13}H_{25}N_4O_4S^+$	333.1591	333.1597	1.5
y_3- NH_3 $C_{17}H_{31}N_6O_4^+$	383.2401	383.2407	1.6
y_3 $C_{17}H_{34}N_7O_4^+$	400.2667	400.2672	1.2
b_4 $C_{18}H_{32}N_5O_5S^+$	430.2119	430.2122	0.7

Peptide 7			
**fragment & formula**	***m/z* calculated**	***m/z* found**	**Error (ppm)**
y_1 / $C_6H_{15}N_4O_2^+$	175.1190	175.1191	0.6
b_2 / $C_9H_{18}N_3O_2S^+$	232.1114	232.1116	0.9
ProArg / $C_{11}H_{20}N_5O_2^+$	254.1612	254.1609	-1.2
y_3 - NH_3 / $C_{17}H_{31}N_8O_4^+$	411.2463	411.2460	-0.7
y_3 / $C_{17}H_{34}N_9O_4^+$	428.2726	428.2728	-0.6
b_4 / $C_{20}H_{37}N_8O_4S^+$	485.2653	485.2651	-0.4

<u>Compound 1</u>: H$_2$N-Cys-Asp-Lys(*h*Arg)-Dab-Dhp-Arg-OH

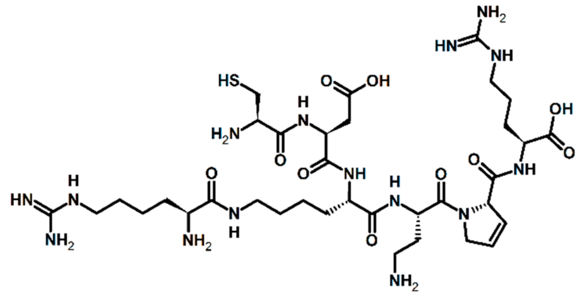

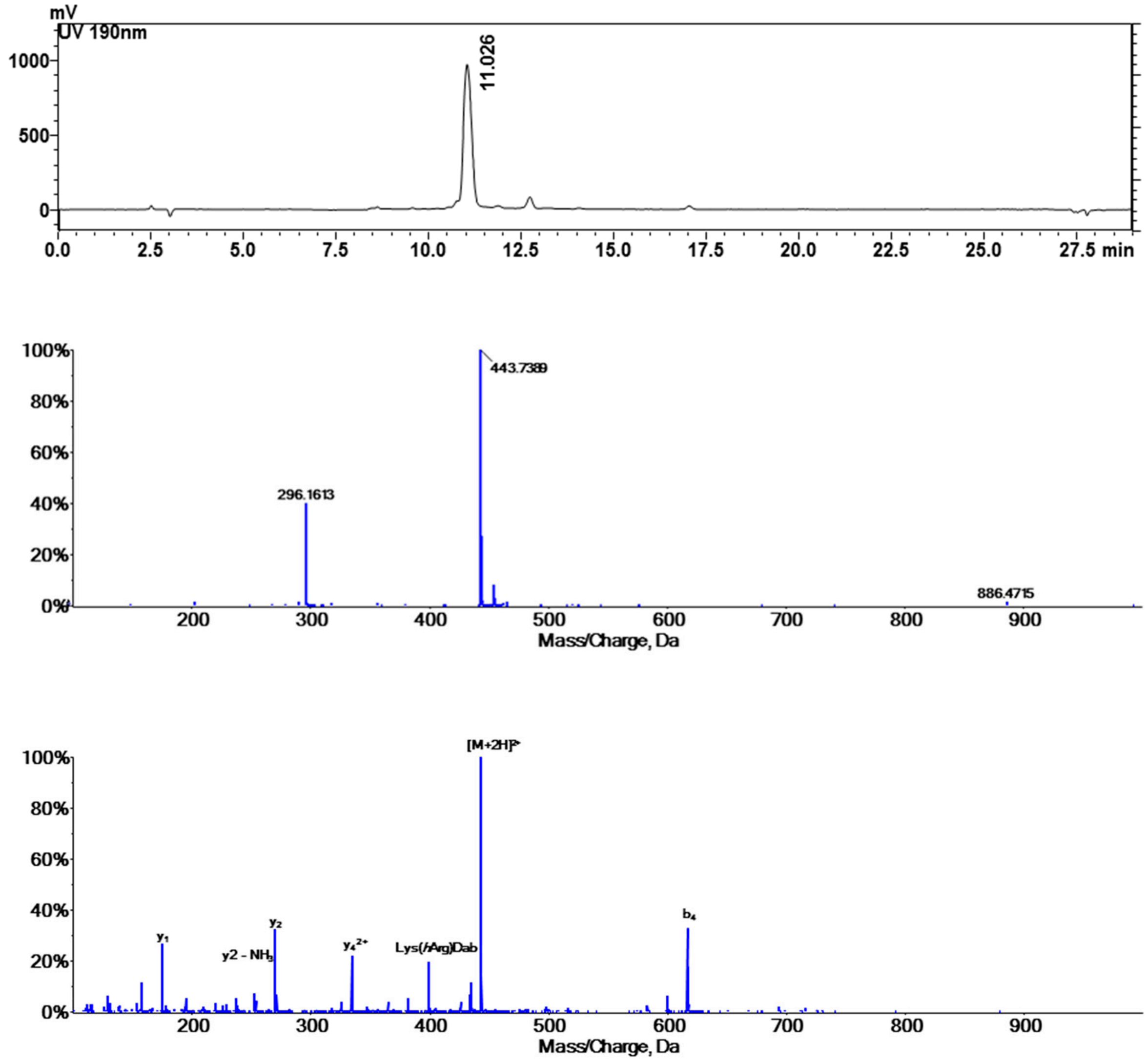

Figure S1. HPLC chromatogram of peptide **1** at 190 nm, HRMS spectrum, and MS/MS fragmentation of $(M+2H)^{2+}$.

Compound 2: H2N-Lys(Cys-Asp-*h*Arg)-Dab-Dhp-Arg-OH

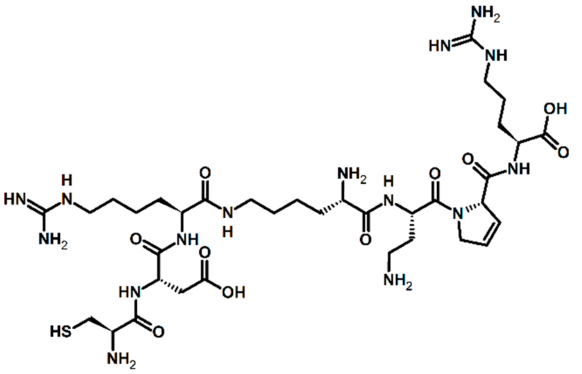

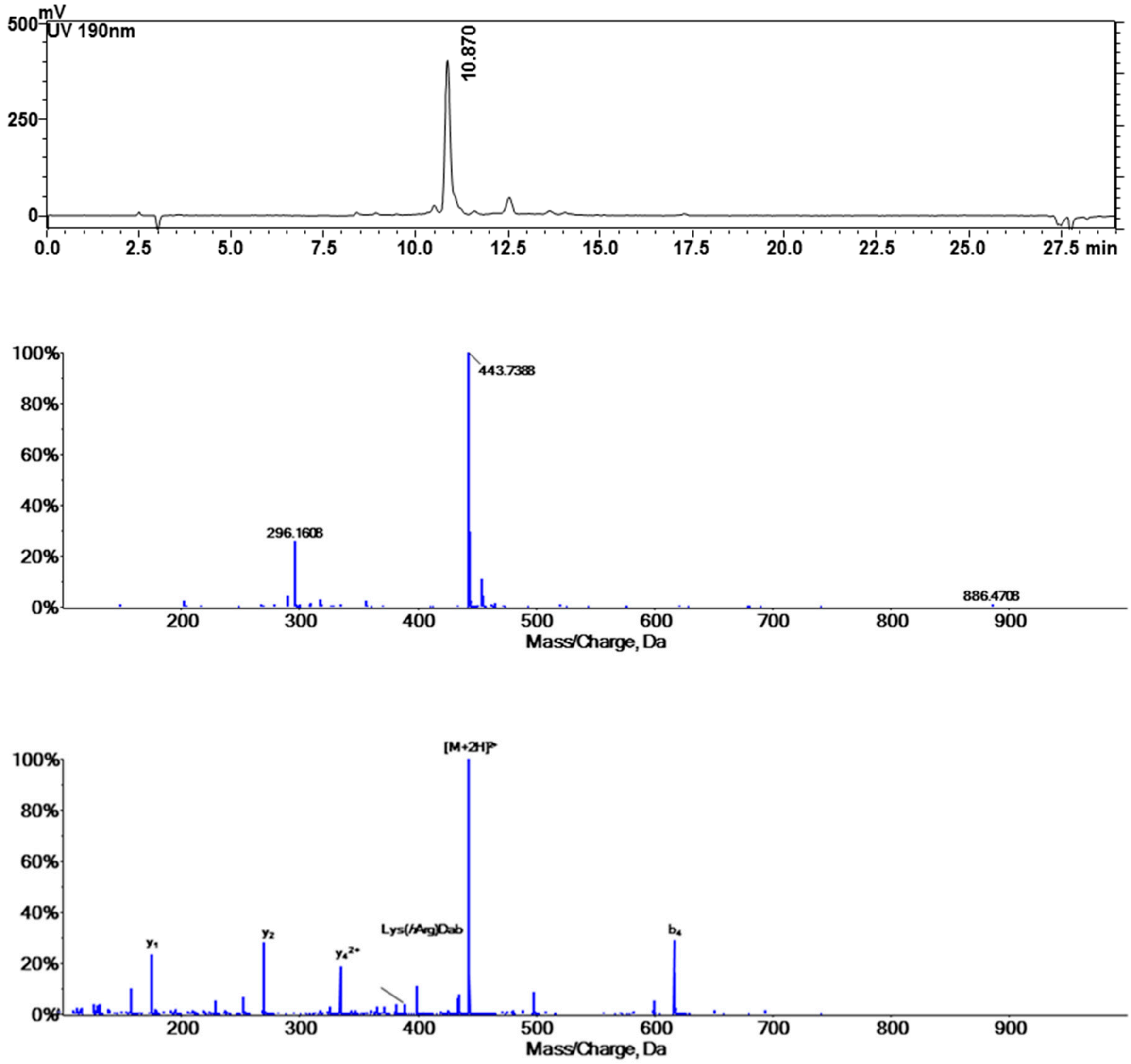

Figure S2. HPLC chromatogram of peptide **2** at 190 nm, HRMS spectrum, and MS/MS fragmentation of $(M+2H)^{2+}$.

<u>Compound 3</u>: H$_2$N-Cys-Lys(*h*Arg)-Dab-Dhp-Arg-OH

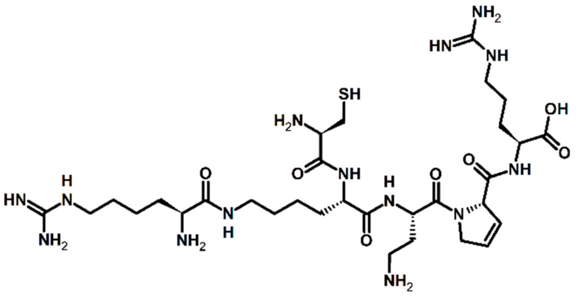

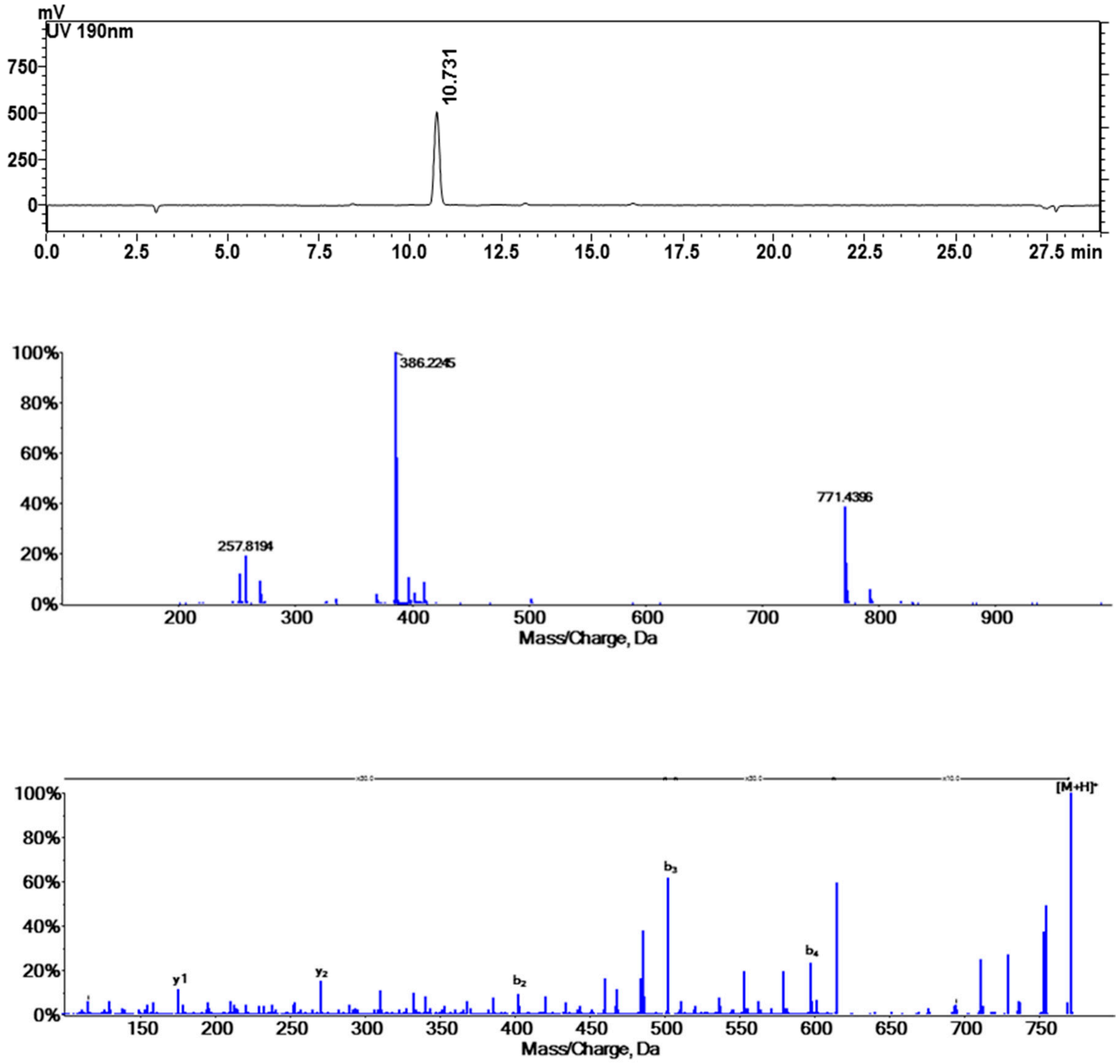

Figure S3. HPLC chromatogram of peptide **3** at 190 nm, HRMS spectrum, and MS/MS fragmentation of (M+H)$^+$.

Compound 4: H_2N-Lys(Cys-*h*Arg)-Dab-Dhp-Arg-OH

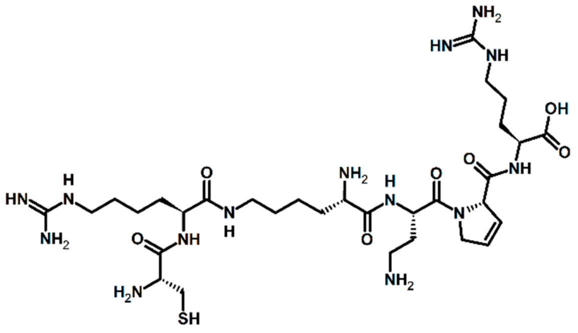

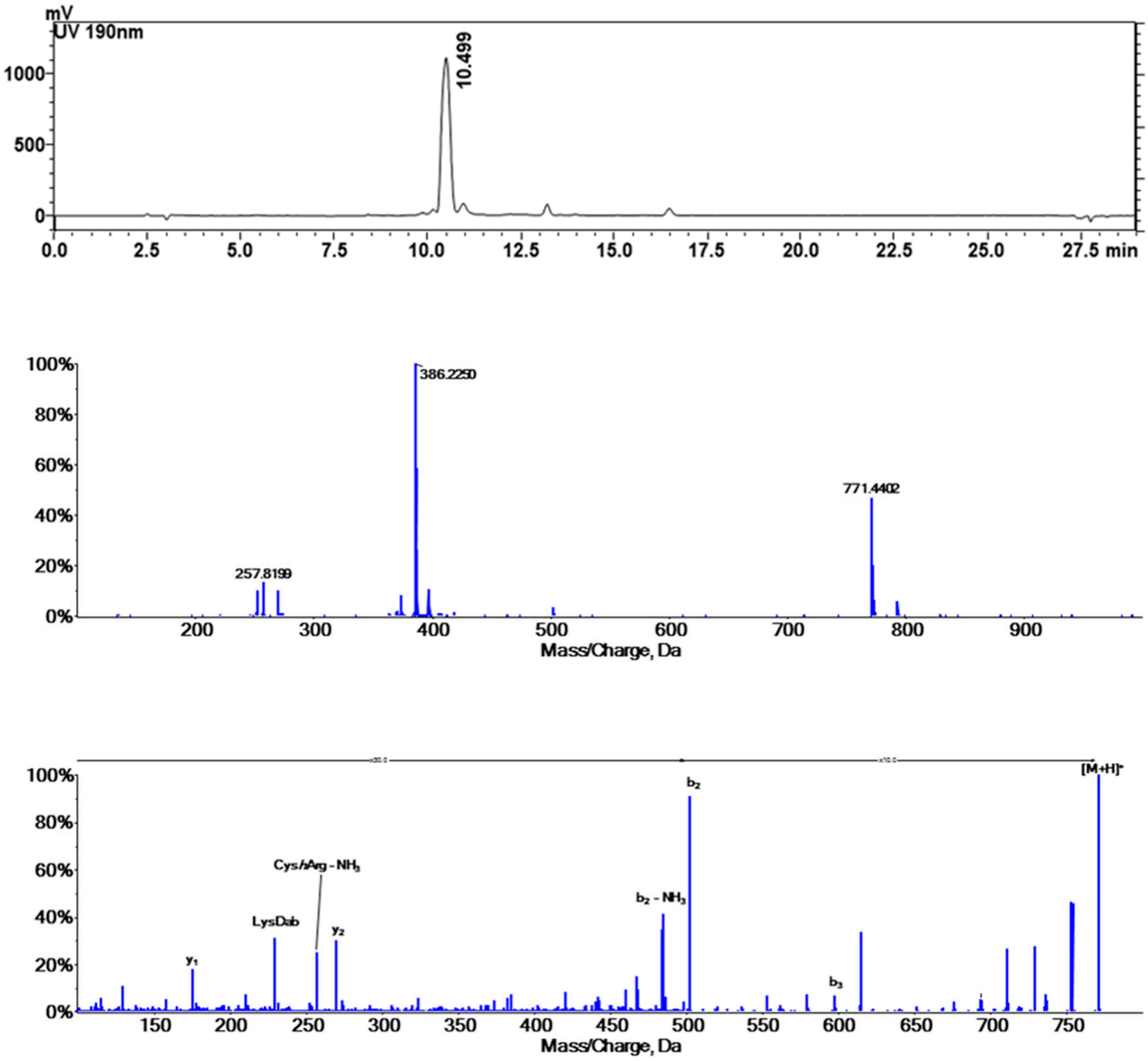

Figure S4. HPLC chromatogram of peptide **4** at 190 nm, HRMS spectrum, and MS/MS fragmentation of $(M+H)^+$.

Compound 5: H2N-Cys-Leu-Pro-Pro-Arg-OH

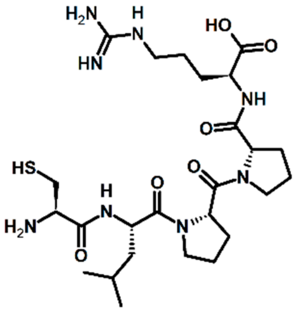

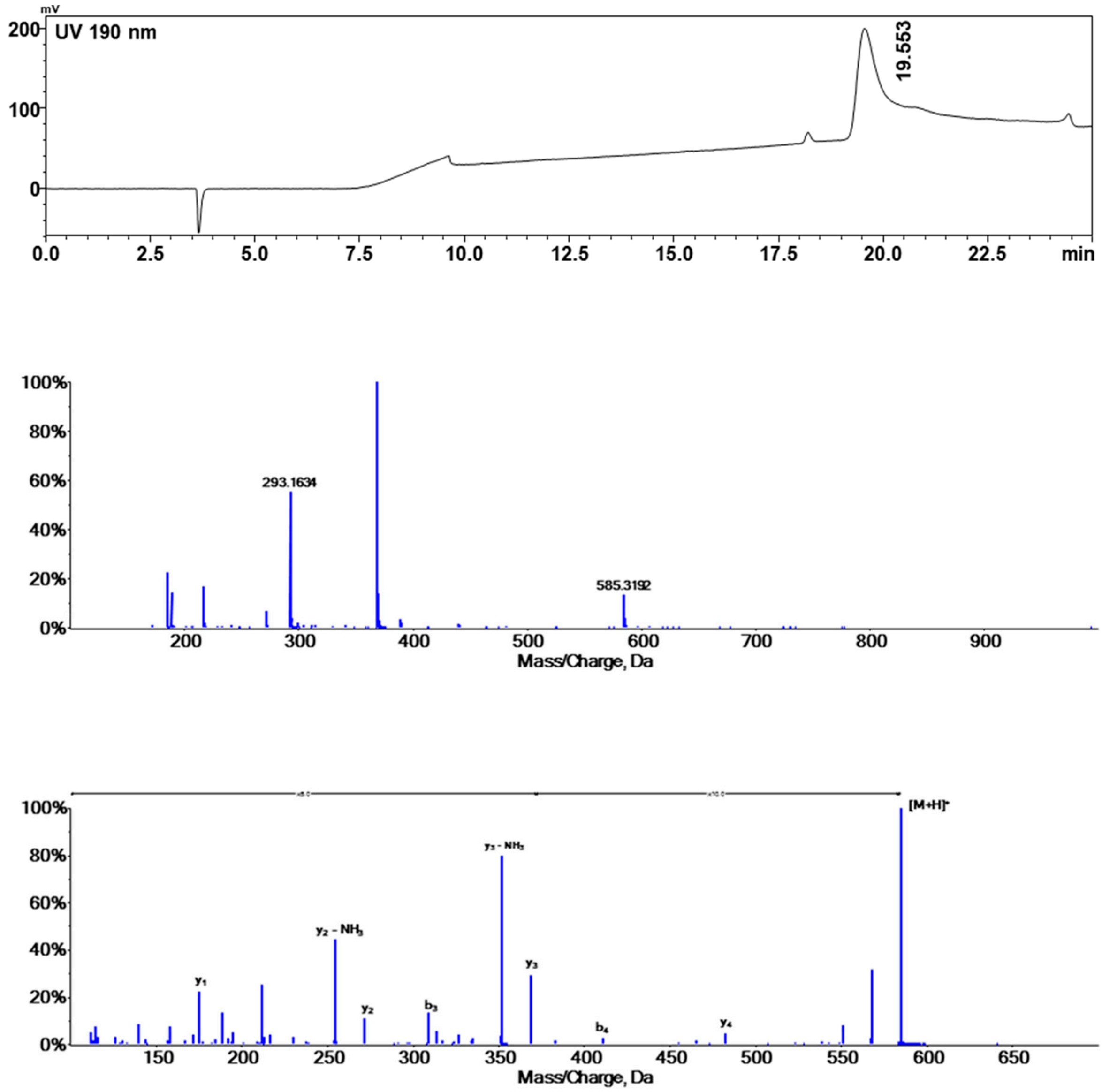

Figure S5. HPLC chromatogram of peptide **5** at 190 nm, HRMS spectrum, and MS/MS fragmentation of $(M+H)^+$.

Compound 6: H_2N- Cys-Thr-Lys-Pro-Arg-OH

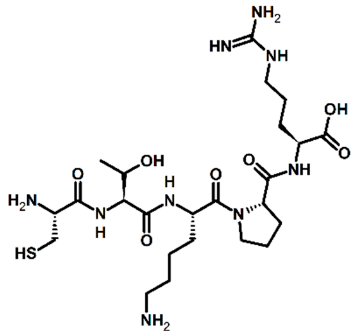

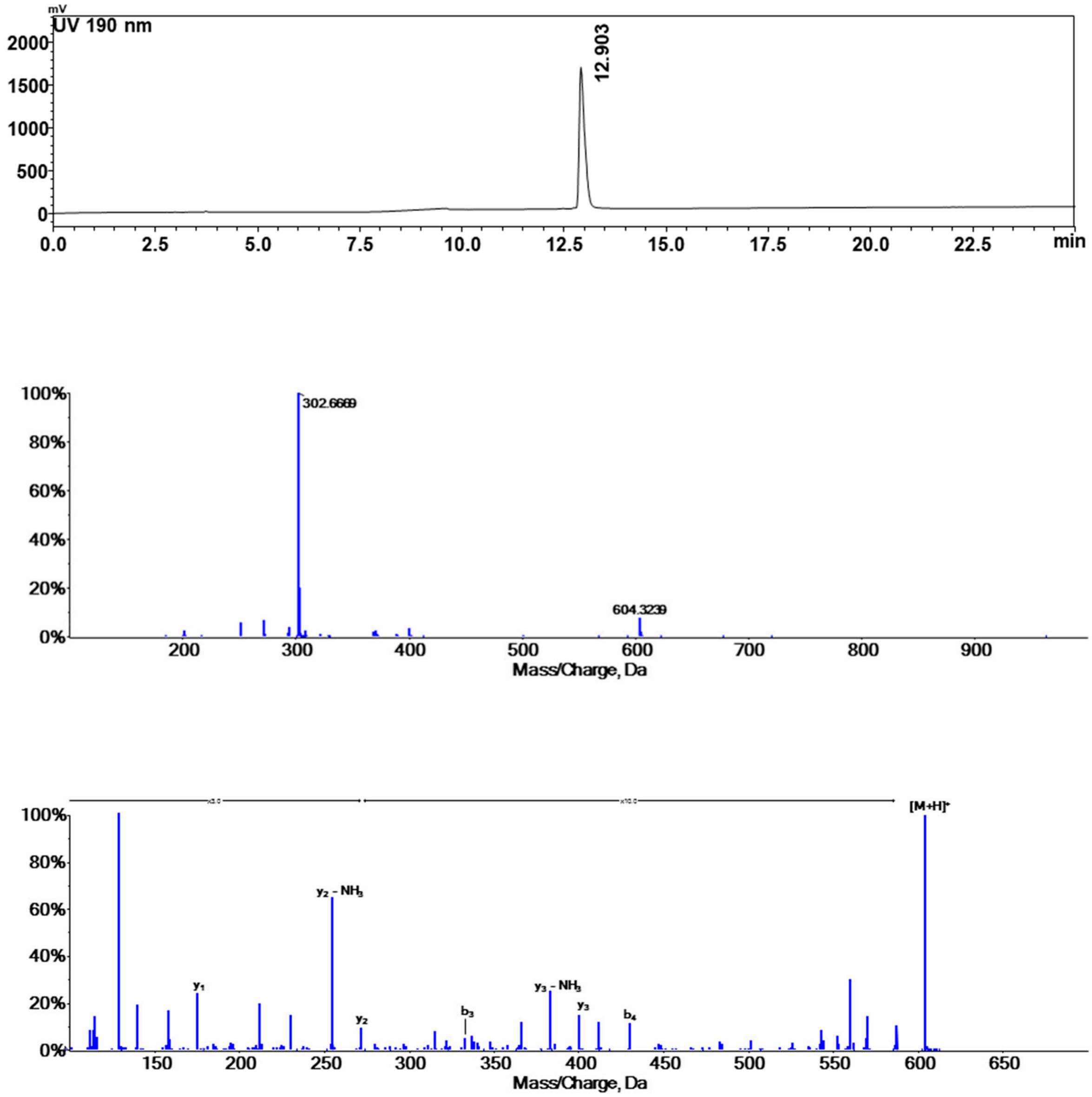

Figure S6. HPLC chromatogram of peptide **6** at 190 nm, HRMS spectrum, and MS/MS fragmentation of $(M+H)^+$.

Compound 7: H_2N- Cys-Lys-Pro-Arg-Arg-OH

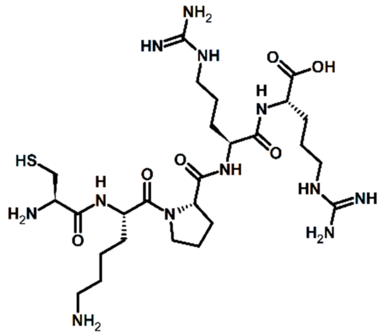

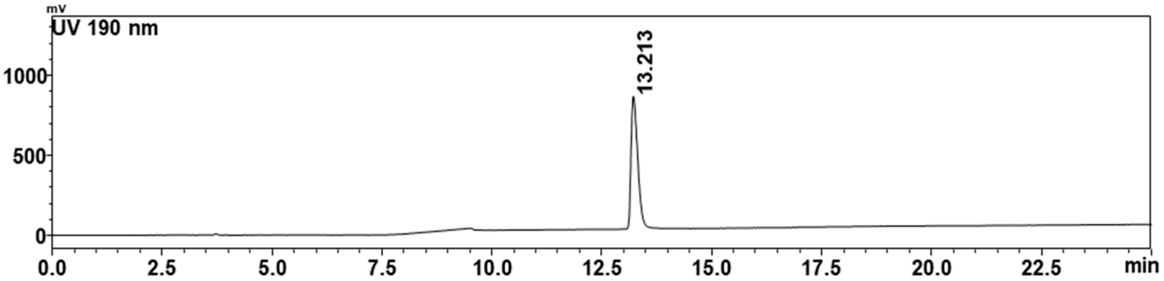

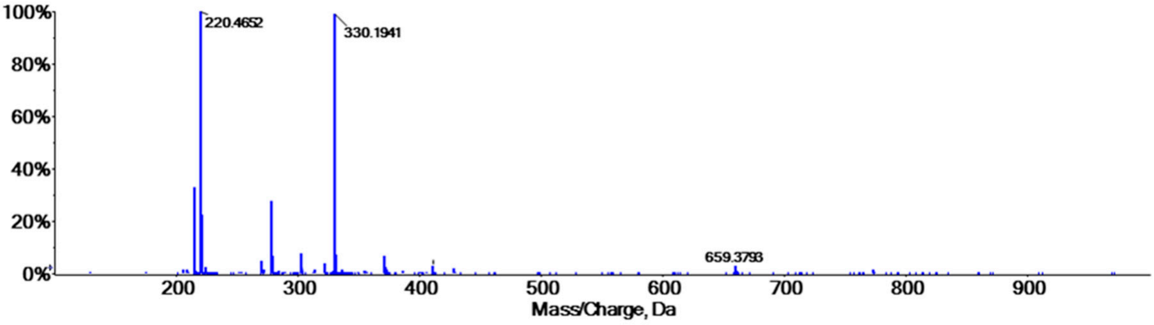

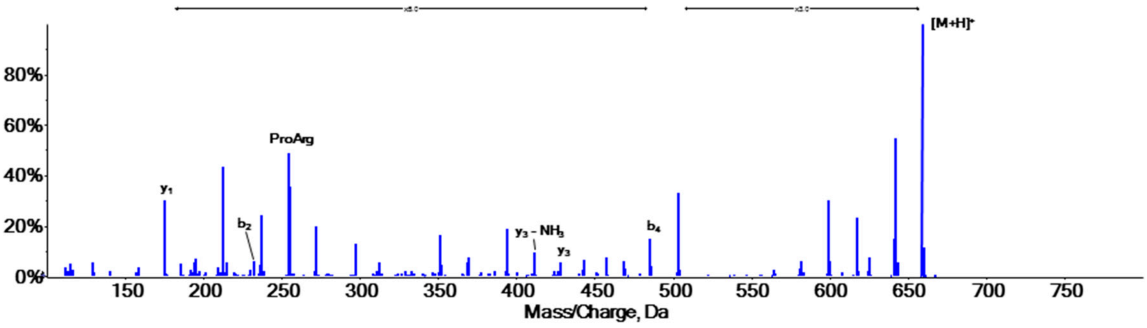

Figure S7. HPLC chromatogram of peptide **7** at 190 nm, HRMS spectrum, and MS/MS fragmentation of $(M+H)^+$.

2. Dose-response curves of synthesized peptides

The concentration-dependent inhibitory dose-curve data were plotted as the percentage inhibition normalized to the controls, with the applied curve fits calculated using GraphPad Prism (Version 5.01, GraphPad software). Data are presented as log(inhibitor) versus a normalized response-variable slope. Error bars represent means +/- SEM for two or three independent experiments. Top and bottom plateau of each curve were constrained to be a constant value equal to the mean of the positive control values and to the mean of the NS values, respectively.

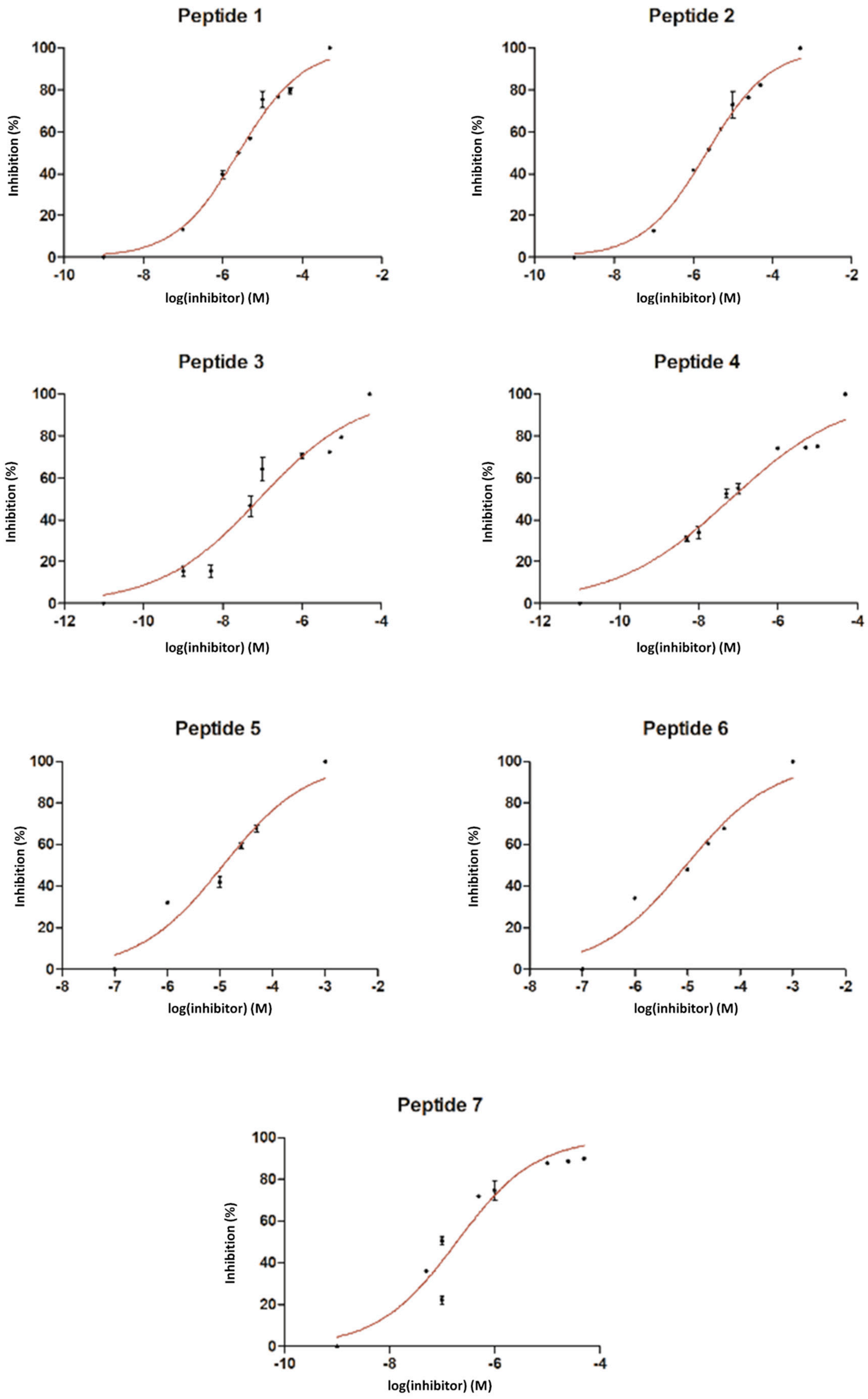

Figure S8. Dose-response curves of peptides **1**-**7**.

Table S4. Confidence intervals (95%) determined for calculated IC_{50}.

Compound	IC_{50} (μM)	95% Confidence Intervals
**1**	2.5	2.1–2.9
**2**	2.0	1.8–2.6
**3**	0.08	0.04–0.16
**4**	0.06	0.04–0.10
**5**	11.4	7.6–17.2
**6**	9.3	6.4–13.6
**7**	0.19	0.13–0.27

3. Analytical data of serum degradation

Analysis of plasma degradation products was carried out by HPLC-ESI-Q-MS with a Prominence HPLC system (binary pump system LC-20AD and autosampler SIL-20AC HT coupled to a SPD-20A UV detector and LCMS-2020 quadrupole mass detector). Chromatographic separation was achieved on a Phenomenex Jupiter Proteo C12 column (250 × 4.6 mm) at 35˚C. Mobile phases consisted of H_2O:TFA (99.95:0.05 v/v, phase A) and ACN:TFA (99.95:0.05 v/v, phase B) at a flow rate of 1.2 mL/min. The eluent was split at a ratio of 1:3 after the UV detector to reduce flow for MS to 0.3 mL/min.

Elution was performed with a gradient as follows: t = 25 min., 0%–25% B. The injection volume was 15 μl. UV spectra were recorded at 190 nm.

The electrospray ionization (ESI) was operated in positive mode. Nitrogen was used as a nebulizing gas, set at 1.5 ml/min, and as a drying gas set at 17 ml/min. The desolvation line and heat block temperature were set at 250°C and 300°C, respectively. Needle voltage was set at +4.5 kV (positive mode) and -4.5kV (negative mode). Detector voltage was set to -1.25kV. Mass spectrometer was used in scan mode in the range of 150-1000 m/z.

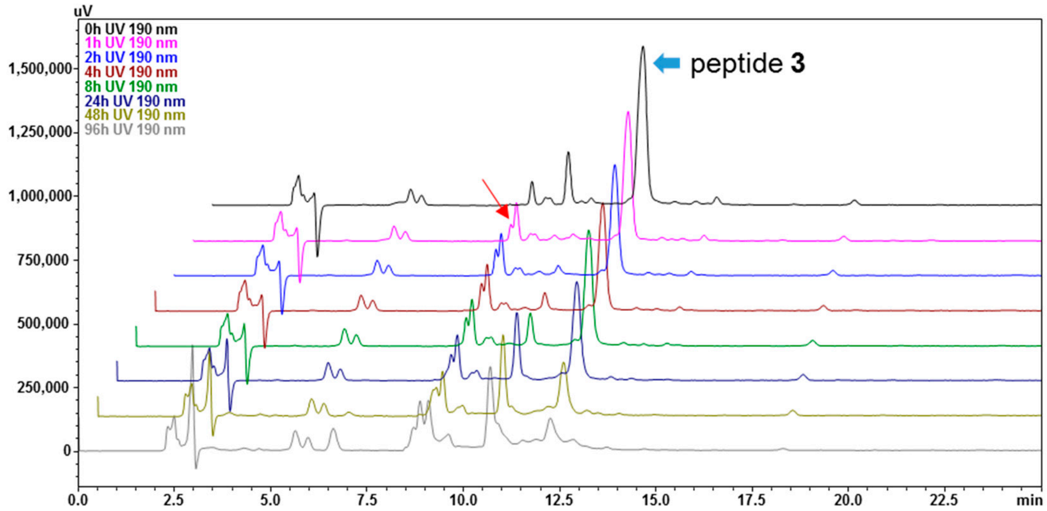

Figure S9. Full chromatogram of peptide **3** after degradation in different time intervals. The red arrow indicates the first metabolite (cleaved cysteine).

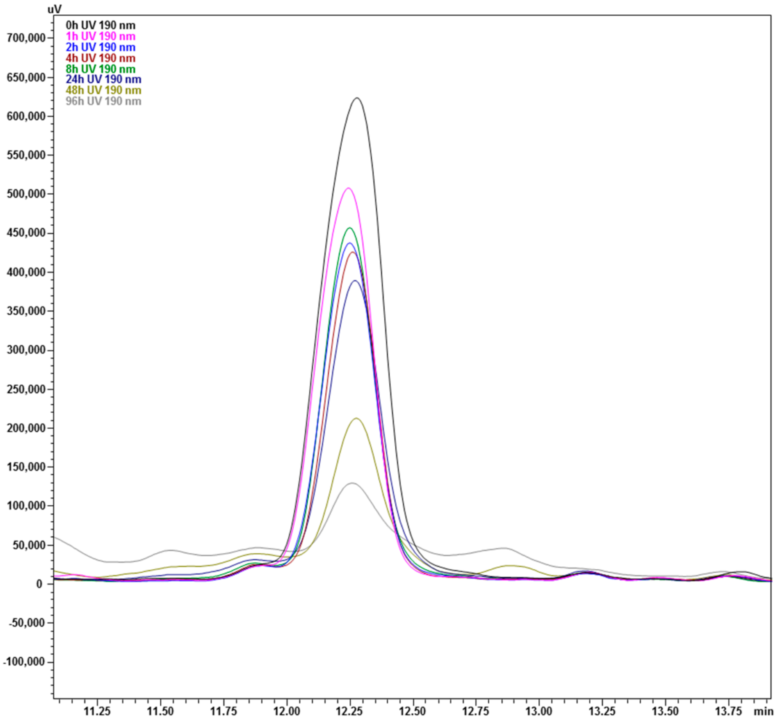

Figure S10. Zoom-in peptide **3** signal after degradation in different time intervals.

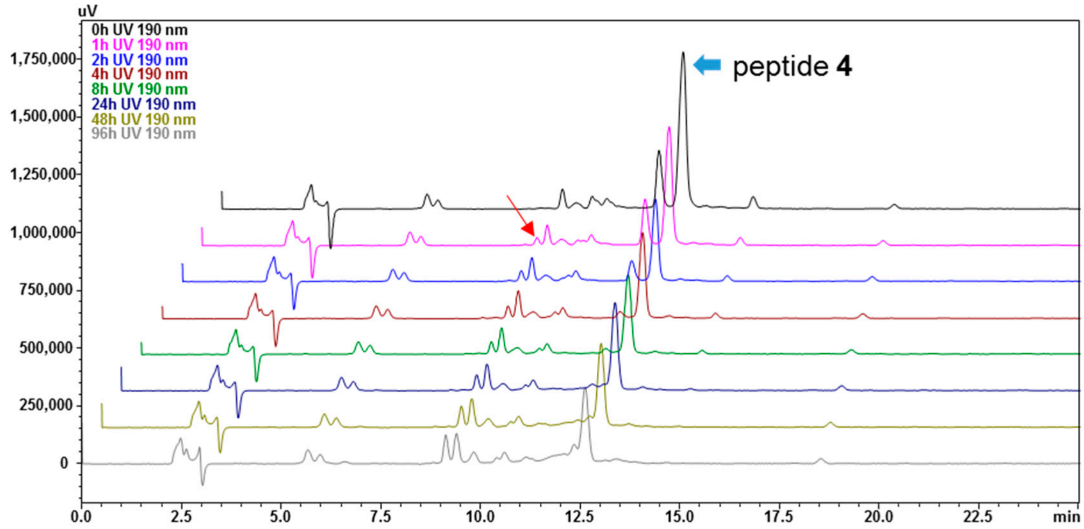

Figure S11. Full chromatogram of peptide **4** after degradation in different time intervals. The red arrow indicates the first metabolite (cleaved Cys).

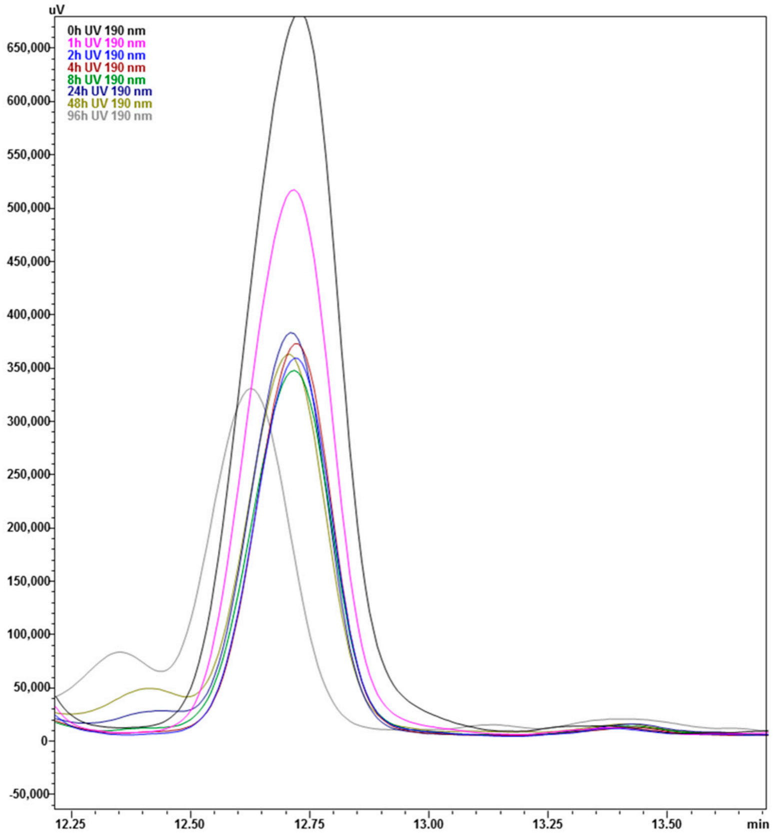

Figure S12. Zoom-in peptide **4** signal after degradation in different time intervals.
